# Identification of the MMP family as therapeutic targets and prognostic biomarkers in the microenvironment of head and neck squamous cell carcinoma

**DOI:** 10.1186/s12967-023-04052-3

**Published:** 2023-03-20

**Authors:** Maohua Liu, Lijuan Huang, Yunling Liu, Sen Yang, Yong Rao, Xiao Chen, Minhai Nie, Xuqian Liu

**Affiliations:** 1 Department of Periodontics & Oral Mucosal Diseases, The Affiliated Stomatology Hospital of Southwest Medical University, Luzhou, Sichuan China; 2 Oral & Maxillofacial Reconstruction and Regeneration of Luzhou Key Laboratory, Luzhou, Sichuan China; 3 Department of Oral and Maxillofacial Surgery, Suining Central Hospital, Suining, Sichuan China; 4 Department of Stomatology Technology, School of Medical Technology, Sichuan College of Traditional Medicine, Mianyang, China; 5 Department of Orthodontics, Mianyang Stomatological Hospital, Mianyang, China

**Keywords:** Head and neck squamous cell carcinoma, MMP, Immunotherapeutic target, Prognostic biomarker

## Abstract

**Background:**

Head and Neck Squamous Cell Carcinoma is a malignant tumor with high morbidity and mortality. The MMP family plays an important role in tumor invasion and metastasis. However, the mechanistic value of the MMP family as a therapeutic target and prognostic biomarker in HNSC has not been fully elucidated.

**Methods:**

Oncomine, UALCAN, GEPIA, cBioportal, GeneMANIA, STRING, DAVID6.8, TRRUST, TIMER and Linkedomics were used for analysis.

**Results:**

The mRNA expression levels of MMP1, MMP3, ILF3, MMP7, MMP9, MMP10, MMP11, MMP12, MMP13 and MMP16 were higher in HNSC than those in normal tissues, while the mRNA expression level of MMP15 was reduced. The relative expression levels of MMP1 and MMP14 were the highest in HNSC tissues. A significant correlation was found between the expression of MMP3, MMP11, MMP25 and the pathological stage of HNSC patients. There was no significant associations between all the MMP family members expression levels and DFS. Increased mRNA levels of MMP1, MMP8 and MMP25 were significantly associated with OS. In addition, we investigated the genetic changes of the MMP family in HNSC and found that all the MMP family members had genetic changes, most of which were amplification and depth loss. In the analysis of neighbor gene network and protein interaction, we found that the MMP family interacted with 25 neighboring genes, except for ILF3, MMP19, MMP20, MMP21, MMP23B, MMP27 and MMP28, other MMP proteins interacted with each other. Functional enrichment analysis showed that the MMP family could be present in the extracellular matrix, regulate peptidase activity, and participate in the catabolism of collagen. Meanwhile, we identified the transcription factor targets and kinase targets of the MMP family and found that ATM and ATR were the two most common kinase targets in the MMP family. We also found a significant correlation between the MMP family expression and immune cell infiltration. Cox proportional risk model analysis showed that macrophages, MMP14, MMP16, and MMP19 were significantly associated with clinical outcomes in HNSC patients.

**Conclusion:**

The MMP family might serve as therapeutic target and prognostic biomarker in HNSC.

## Introduction

Head and neck squamous cell carcinoma (HNSC) is one of the most common malignant tumors, which originates in the oral and nasopharynx, larynx, or pharynx. It is associated with high morbidity and mortality and research has shown that global incidence is about 600,000 cases and accounting for around 380,000 deaths every year [1]. The treatment of HNSC has a poor prognosis, with more than 50% of locally advanced cases recurrent after surgery or chemotherapy [2]. Targeting therapy is a critically important mode of cancer therapy research, most of all, to overcome the immunosuppressive tumor microenvironment. The anti-PD1/PD-L1 checkpoint inhibitors are the first drugs that have shown any survival benefit for the treatment on platinum-refractory recurrent/metastatic (R/M) HNSC, and the PD-L1 can improve Overall Survival (OS) and quality of life [3]. A study has explored that the role of CD244 of the immunosuppressive environment assessed in HNSC and assessed its therapeutic potential. Compared with healthy tissues, the CD244 expression shows significant increased expression from HNSC tissues, which correlated with PD1 expression [4]. In HNSC, CMTM6, a regulator of PD-L1 expression, is overexpressed. Gene resection of CMTM6 can reduce the expression of PD-L1 and inhibit the proliferation and migration of HNSC cancer cells, so CMTM6 can be used as a targeted therapeutic point for HNSC [5].

Although biomarkers such as PD-L1 has become the focus of HNSC immunotherapy checkpoint inhibition, this is only part of the HNSC cell immunosuppression biomarkers. Matrix metalloproteinases are enzymes that degrade various protein components of the extracellular Matrix. In HNSC, the MMP family degrade the extracellular Matrix and damage the basement membrane, playing a key role in tumor invasion and metastasis. The MMP family are linked to tumor proliferation, differentiation and angiogenesis, because they can activate growth factors and enhance angiogenesis [6]. The MMP family can be used as therapeutic targets and prognostic biomarkers for HNSC based on its role in the disease. Some scholars used sesamin extracted from peppercorns bark sesame oil to regulate MMP2, thus inhibiting the migration and invasion of HNSC [7]. Some studies have shown that mulberry leaf extract can inhibit MMP2 and MMP9 activities and inhibit HNSC migration and invasion [8].

Although many studies have used the MMP family as a therapeutic target and prognostic marker, the expression levels of various members of the MMP family are different in HNSC cells, and HNSC cells of different cell lines can also express different biomarkers and MMP. This article aims to study the expression, prognosis, mutation and protein interaction, functional enrichment, related signaling pathways and kinase targets of the MMP family in HNSC, to accurately explore that the MMP family can be used as therapeutic targets and prognostic biomarkers for HNSC.

## Materials and methods

### Oncomine

Oncomine, as a bioinformatics database that can collect and analyze cancer transcriptome data, provides powerful genome-wide expression analysis [9]. The identification of key genomic biomarkers is conducive to the diagnosis, prognosis and treatment of diseases [10]. In this study, data was extracted to evaluate the expression of the MMP family in HNSC, where *P* < 0.05, FC ≥ 2, and the top 10% of genes were the significance thresholds, and t test was used to analyze the expression differences of the MMP family in HNSC.

### UALCAN

By obtaining data from TCGA, UALCAN can be used not only to assess the expression of protein-coding genes, but also to conduct in-depth analysis of clinical data in 33 cancers [11]. In this study, expression data of the MMP family was obtained through “Expression Analysis”module and “KIRC” dataset, and t test was used for analysis, with *P* < 0.05 as the significance threshold.

### GEPIA

GEPIA is an analysis tool based on TCGA and GTEx data, which contains RNA sequence expression data of 9736 tumors and 8587 normal tissue samples [12]. In this study, GEPIA single gene analysis was used to analyze the difference of mRNA expression between tumor tissue and normal tissue, pathological staging analysis and prognosis analysis of the MMP family. The HNSC dataset was used to analyze the MMP family by polygene comparison. T test analysis was used, with *P* < 0.05 as the significant threshold, and Kaplan–Meier curve was used for prognosis analysis.

### cBioportal

cBioportal is a platform for exploring, visualizing, and analyzing multi-dimensional cancer genomic data. cBioportal contains over 200 cancer genomics studies from the TCGA database [13]. In this study, genetic alteration, co-expression and network modules of the MMP family were obtained from cBiopartal based on TCGA database. A total of 564 HNSC specimens were analyzed.

### GeneMANIA

GeneMANIA, based on genomic, proteomic and gene functional data, aims to provide information on protein-genetic interactions, pathways, co-expression, co-localization, and similarity of protein domains of submitted genes [14].

### STRING

STRING aims to collect, score, and integrate protein–protein interaction data from all publicly available sources, and to predict and supplement these data through potential function calculations [15]. In this study, PPI network analysis was performed on the different-expressed the MMP family to explore the interaction between this family and STRING.

### DAVID6.8

DAVID6.8 provides a method for elucidating the biological functions of the submitted genes [16]. In this study, GO enrichment analysis and KEGG pathway enrichment analysis of the MMP family and adjacent genes were isolated from DAVID6.8, including BP, CC, MF.

### TRRUST

TRRUST contains 8,444 TF regulatory relationships of 800 human transcription factors, which can provide how these interactions are regulated [17], and is an intuitive and reliable tool for human transcriptional regulatory networks.

### TIMER

TIMER provides a systematic evaluation of different immune cell infiltrates and their clinical effects [18]. In this study, the gene module was used to evaluate the correlation between the MMP family levels and immune cell infiltration, and the survival module was used to evaluate the correlation between clinical outcomes and immune cell infiltration and the MMP family.

### Linkedomics

Linkedomics contains a multiomics data analysis of 32 TCGA cancer types [19]. In this study, the biological analysis of the enrichment of the MMP family kinase target was carried out using LinkInterpreter. GSEA was used for at least 3 genes and 500 simulations in the HNSC datasets. Spearman correlation test was adopted, and *P* < 0.05 was the significant threshold.

### Statistical analysis

The expression difference of the MMP family in HNSC was analyzed using the t test. The R software and Graphpad prism 9.0 software were used for statistical analysis of the data obtained from each database, and the results were visualized. Kaplan–Meier curve and log-rank test were used to analyze whether the transcription level of the MMP family was significantly correlated with disease-free survival. For statistical correlation, Spearman correlation coefficient was used according to requirements, with *P* < 0.05 as the threshold of significance.

## Results

### The MMP family mRNA expression profiles in various cancers and different HNSC datasets

**Fig. 1:**
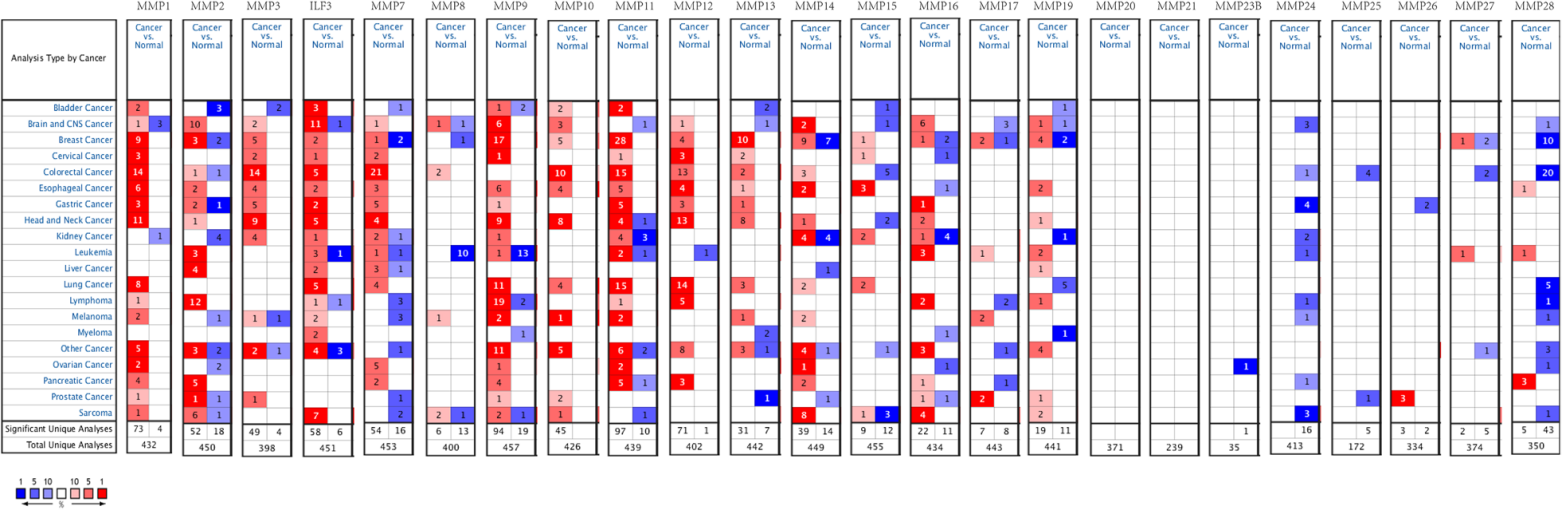
mRNA levels of the MMP family in HNSC

We used Oncomine database to investigate mRNA expression of the MMP family in different tumor types and to detect their levels in different HNSC datasets. The database contained mRNA expression of 24 MMP family members in 20 tumors. MMP1 mRNA expression was significantly different in 77 studies. In 73 of 77 studies, 16 of 20 tumors were observed to have higher levels of mRNA expression than normal tissue, and only 2 of the remaining 4 studies had lower levels of mRNA expression than normal tissue; The mRNA expression levels of MMP2 and MMP7 in 13 of 20 tumors were higher than those in normal tissues. The mRNA expression levels of MMP3, MMP10, MMP12 and MMP16 in 11 tumors were higher. The mRNA expression levels of ILF3 and MMP9 were higher than those of normal tissues. Among 439 studies of MMP11, 107 studies showed significant differences and among 97 studies, 15 tumor mRNA expression levels were higher than normal tissues. MMP13 mRNA was highly expressed in 9 kinds of tumors. MMP14 was highly expressed in 12 tumors. MMP19 was highly expressed in 10 tumors. The expression of MMP15, MMP17, MMP24 and MMP28 were low in most of the 20 tumors. The mRNA expression levels of MMP8, MMP20, MMP21, MMP23B, MMP25, MMP26 and MMP27 were not significantly different among different tumors. In 24 HNSC data sets, MMP1, MMP3, ILF3, MMP7, MMP9, MMP10, MMP11, MMP12, MMP13, and MMP16 were expressed at higher levels in most tumor tissues than in normal tissues, while MMP15 was expressed at lower level (Fig. 1).

Detailed data on independent HNSC datasets with significant differences in mRNA expression was listed in Oncomine (Table 1) [20–25]. From the table, MMP1 and MMP10 were highly expressed in oral squamous cell carcinoma. The expression of MMP3 and ILF3 was up-regulated in tongue squamous cell carcinoma. MMP7, MMP9, MMP11, MMP12 and MMP13 were all up-regulated in head and neck squamous cell carcinoma. Compared with normal tissues, MMP16 expression was up-regulate in thyroid papillary carcinoma. MMP15 was down-regulated in all 14 HNSC tumors, including Buccal Mucosa Squamous Cell Carcinoma, Floor of Mouth Squamous Cell Carcinoma, Gingival Squamous Cell Carcinoma, Glottis Squamous Cell Carcinoma, Hard Palate Squamous Cell Carcinoma, Lip Squamous Cell Carcinoma, Maxillary Sinus Squamous Cell Carcinoma, Oral Cavity Squamous Cell Carcinoma, Oropharyngeal Squamous Cell Carcinoma, Postcricoid Squamous Cell Carcinoma, Soft Palate Squamous Cell Carcinoma, Supraglottic Squamous Cell Carcinoma, Tongue Squamous Cell Carcinoma, Tonsillar Squamous Cell Carcinoma.

**Table 1:** The mRNA levels of the MMP family in different types HNSC tissues and normal tissues at transcriptome level

TLR	Type	Fold change	*P* value	T test	References
MMP1	Oral Cavity Squamous Cell Carcinoma (57)	86.331	5.71E−44	30.373	Peng et al. [20]
MMP3	Tongue Squamous Cell Carcinoma (26)	10.568	1.06E−12	10.471	Ye et al. [21]
ILF3	Tongue Squamous Cell Carcinoma (3)	2.019	4.38E−5	7.408	Kuriakose et al. [22]
MMP7	Head and Neck Squamous Cell Carcinoma4 (41)	7.534	7.00E−14	10.461	Ginos et al. [23]
MMP9	Head and Neck Squamous Cell Carcinoma5 (41)	11.764	7.07E−26	19.530	Ginos et al. [23]
MMP10	Oral Cavity Squamous Cell Carcinoma (57)	25.608	5.49E−32	20.959	Peng et al. [20]
MMP11	Head and Neck Squamous Cell Carcinoma (34)	10.020	3.88E−6	9.649	Cromer et al. [24]
MMP12	Head and Neck Squamous Cell Carcinoma (41)	15.603	5.50–24	17.721	Ginos et al. [23]
MMP13	Head and Neck Squamous Cell Carcinoma (41)	15.206	6.80E−12	8.766	Ginos et al. [23]
MMP16	Thyroid Gland Papillary Carcinoma (14)	2.127	2.26E−4	4.703	Vasko et al. [25]
MMP15	Buccal Mucosa Squamous Cell Carcinoma (2)				Kuriakose et al. [22]
	Floor of Mouth Squamous Cell Carcinoma (1)				
	Gingival Squamous Cell Carcinoma (2)				
	Glottis Squamous Cell Carcinoma (2)				
	Hard Palate Squamous Cell Carcinoma (1)				
	Lip Squamous Cell Carcinoma (1)				
	Maxillary Sinus Squamous Cell Carcinoma (1)				
	Oral Cavity Squamous Cell Carcinoma (2)				
	Oropharyngeal Squamous Cell Carcinoma (1)				
	Postcricoid Squamous Cell Carcinoma (1)				
	Soft Palate Squamous Cell Carcinoma (2)				
	Supraglottic Squamous Cell Carcinoma (2)				
	Tongue Squamous Cell Carcinoma (3)				
	Tonsillar Squamous Cell Carcinoma (1)				

**Fig. 2:**
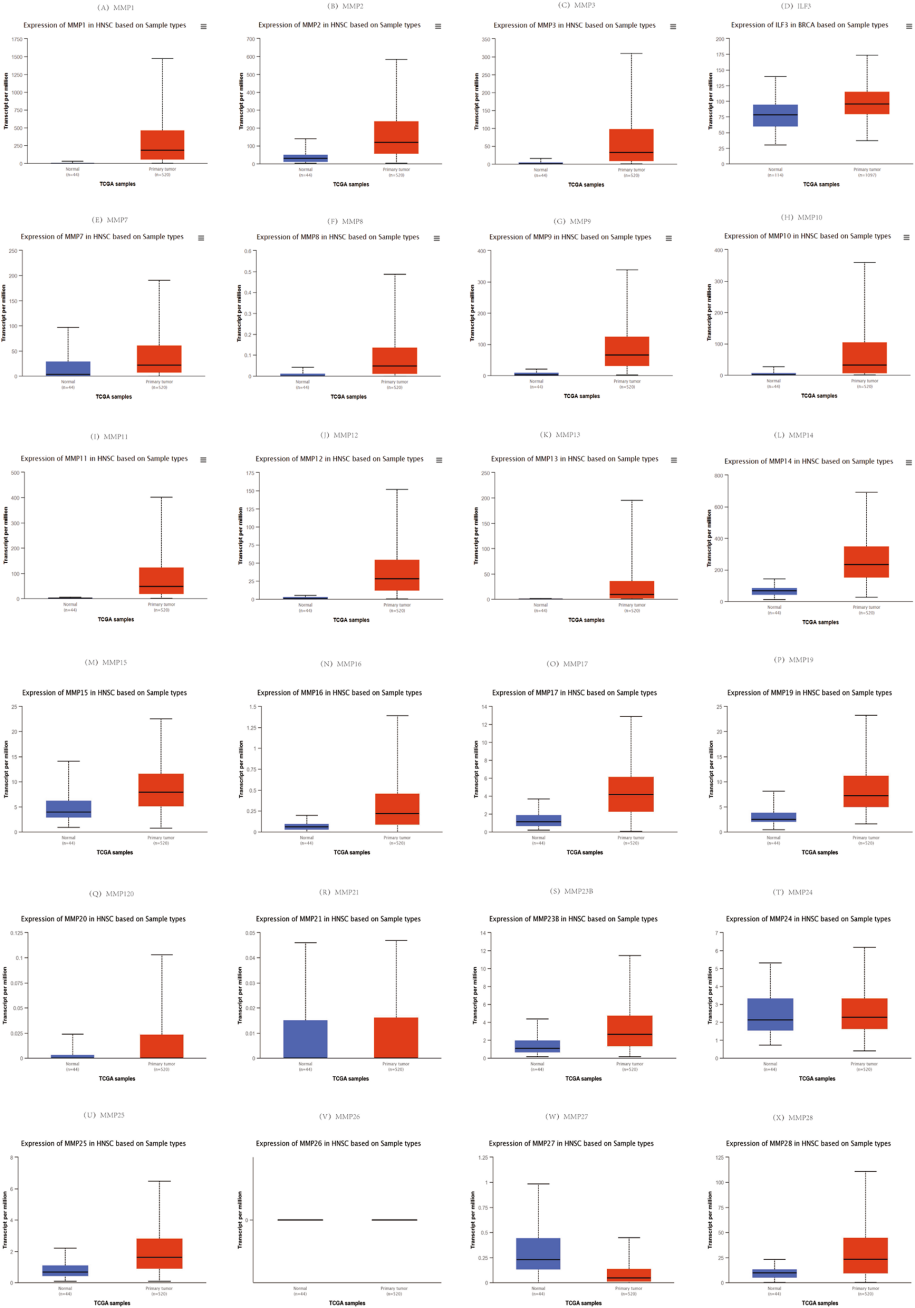
The transcription of the MMP family in HNSC

**Fig. 3:**
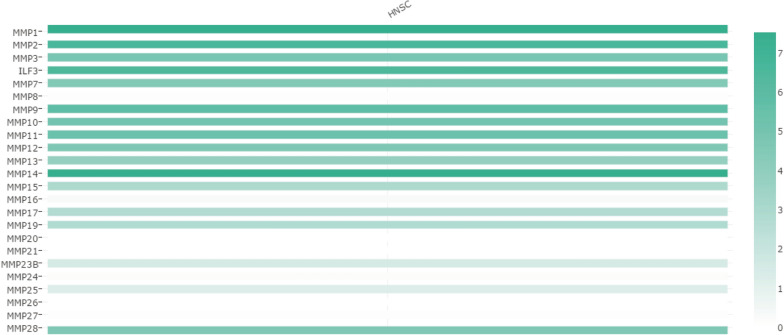
The relative level of the MMP family in HNSC

According to the UALCAN analysis, MMP2 (*P* = 3.81E−04), MMP3 (*P* = 1.62E−12), ILF3 (*P* = 1.62E−12), MMP8 (*P* = 3.33E−08), MMP9 (*P* = 1.62E−12), MMP10 (*P* = 9.30E−10), MMP12 (*P* = 1.62E−12), MMP14 (*P* = 1.62E−12), MMP15 (*P* = 1.28E−06), MMP16 (*P* = 1.35E−04), MMP17 (*P* < 1E−12), MMP19 (*P* = 3.33E−15), MMP20 (*P* = 6.96E−04), MMP23B (*P* = 1.66E−03), MMP25 (*P* = 3.90E−07) and MMP28 (*P* < 1E−12) mRNA transcription levels were higher than those of normal tissues. The transcription level of MMP27 (*P* = 5.64–03) was significantly decreased (Fig. 2). We also compared the relative expression levels of the MMP family in HNSC, and found that MMP1 and MMP14 were the highest relative expression levels among all the MMP family members in HNSC tissues (Fig. 3).

**Fig. 4:**
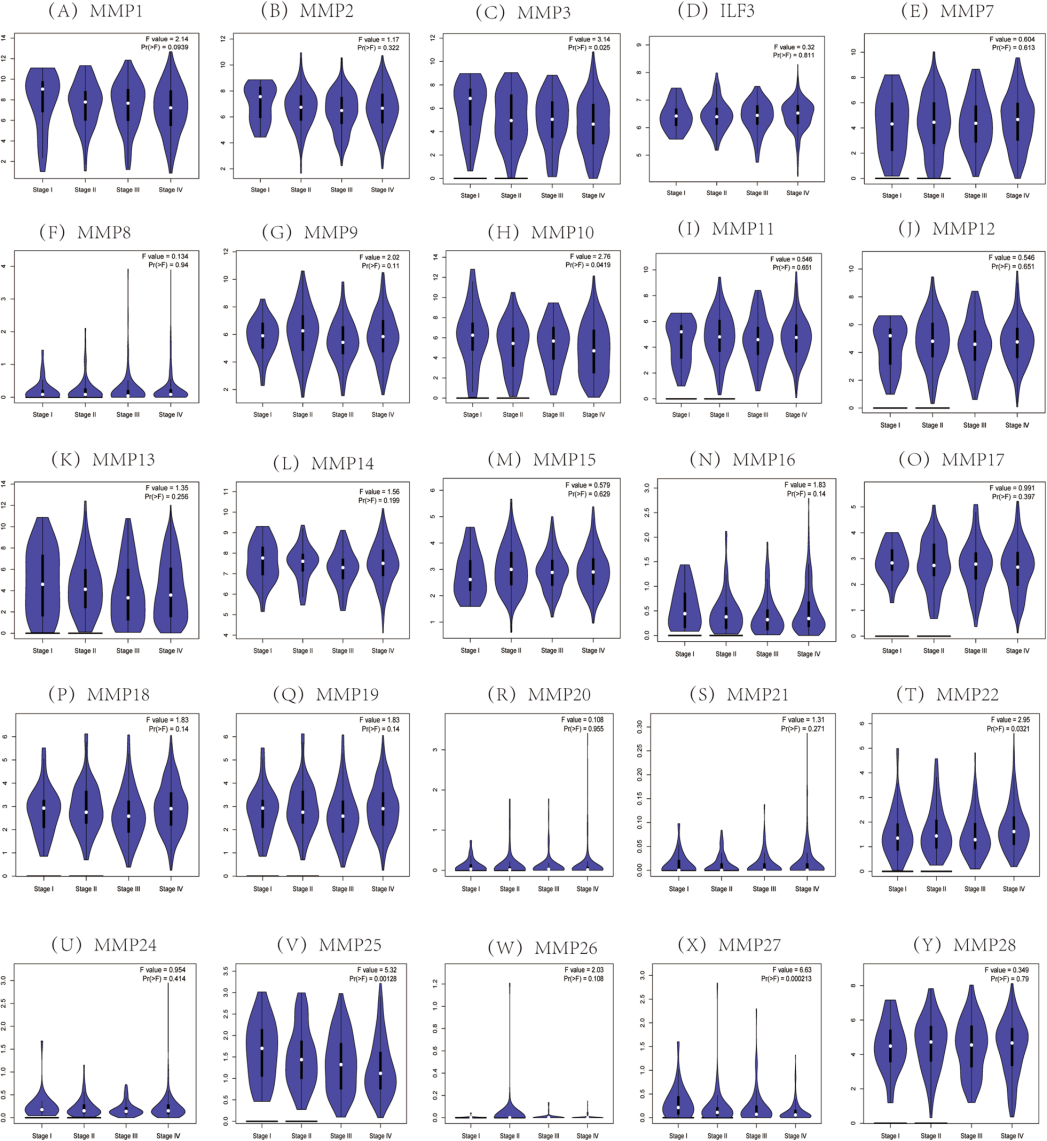
Correlation between the MMP family expression and pathological stage of HNSC patients

We then evaluated the correlation between the MMP family expression and pathological stage in HNSC patients, and found that the expression of MMP3 (F = 3.14, *P* = 0.025), MMP11 (F = 3.25, *P* = 0.025) and MMP25 (F = 5.32, *P* = 0.001) were significantly correlated with pathological stage (Fig. 4). With the development of HNSC, the expression of MMP3 and MMP11 increased, and the expression of MMP25 decreased significantly.

### Prognostic value of the MMP family in patients with head and neck squamous cell carcinoma

**Fig. 5:**
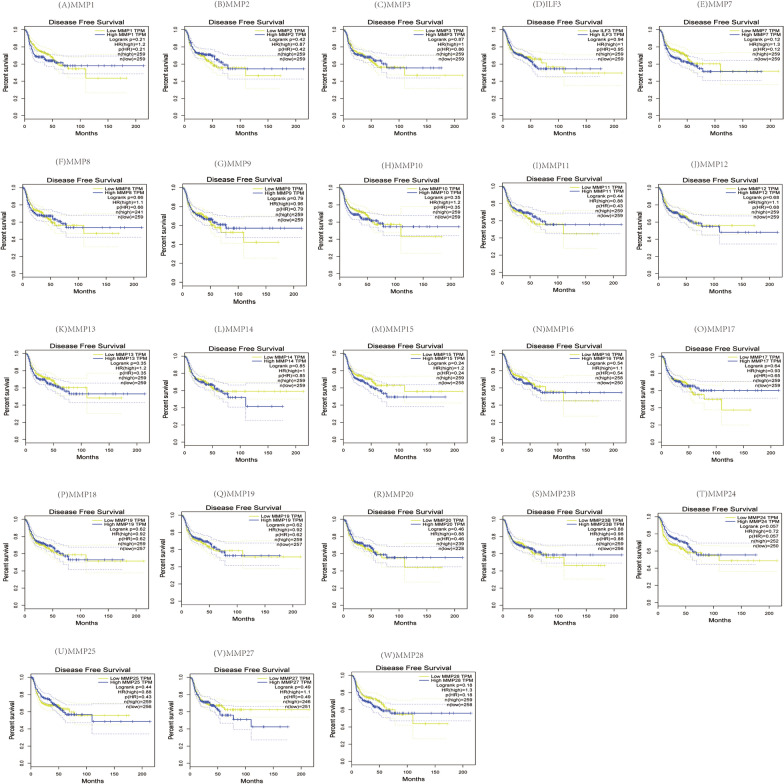
The prognostic value of the MMP family in HNSC patients in the disease free survival curve

**Fig. 6:**
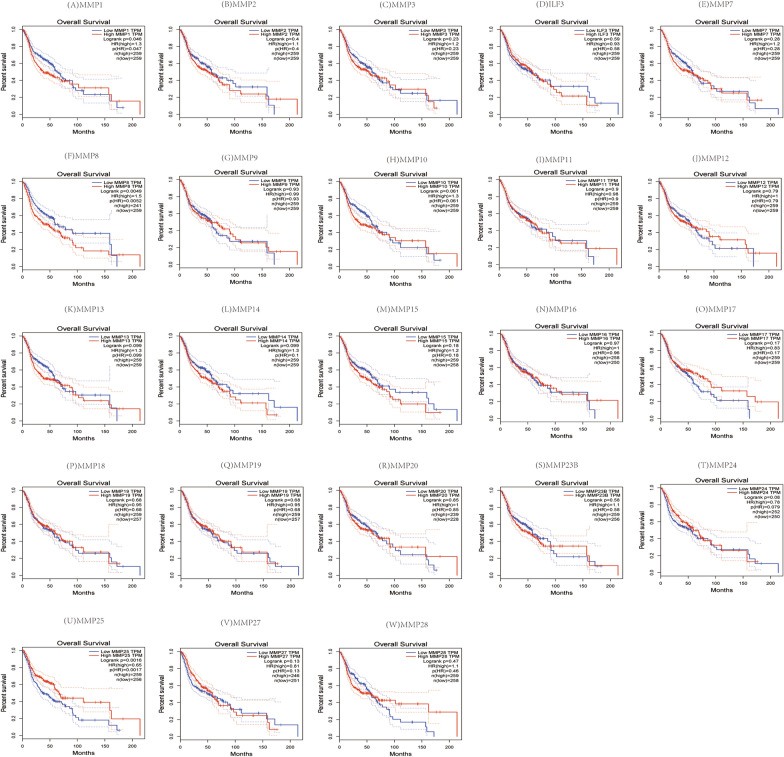
The prognostic value of the MMP family in HNSC patients in the overall survival curve

To assess the value of the MMP family in the progression of HNSC, we used GEPIA to evaluate the association of differential expression of the MMP family with clinical outcomes. The Kaplan–Meier curve and log-rank test analysis revealed that there was no significant correlation between high and low transcription levels of all the MMP family members with disease-free survival rate (Fig. 5). It was found that increased mRNA level of MMP1 (*P* = 0.045), When exploring the correlation between the MMP family expression and overall survival in HNSC patients. MMP8 *(P* = 0.005) and MMP25 (*P* = 0.002) were significantly correlated with overall survival (*P* < 0.05), while no significant difference was found in other MMP (Fig. 6).

### Relationship between genetic alteration, co-expression and protein/gene interaction of the MMP family in patients with head and neck squamous cell carcinoma

**Fig. 7:**
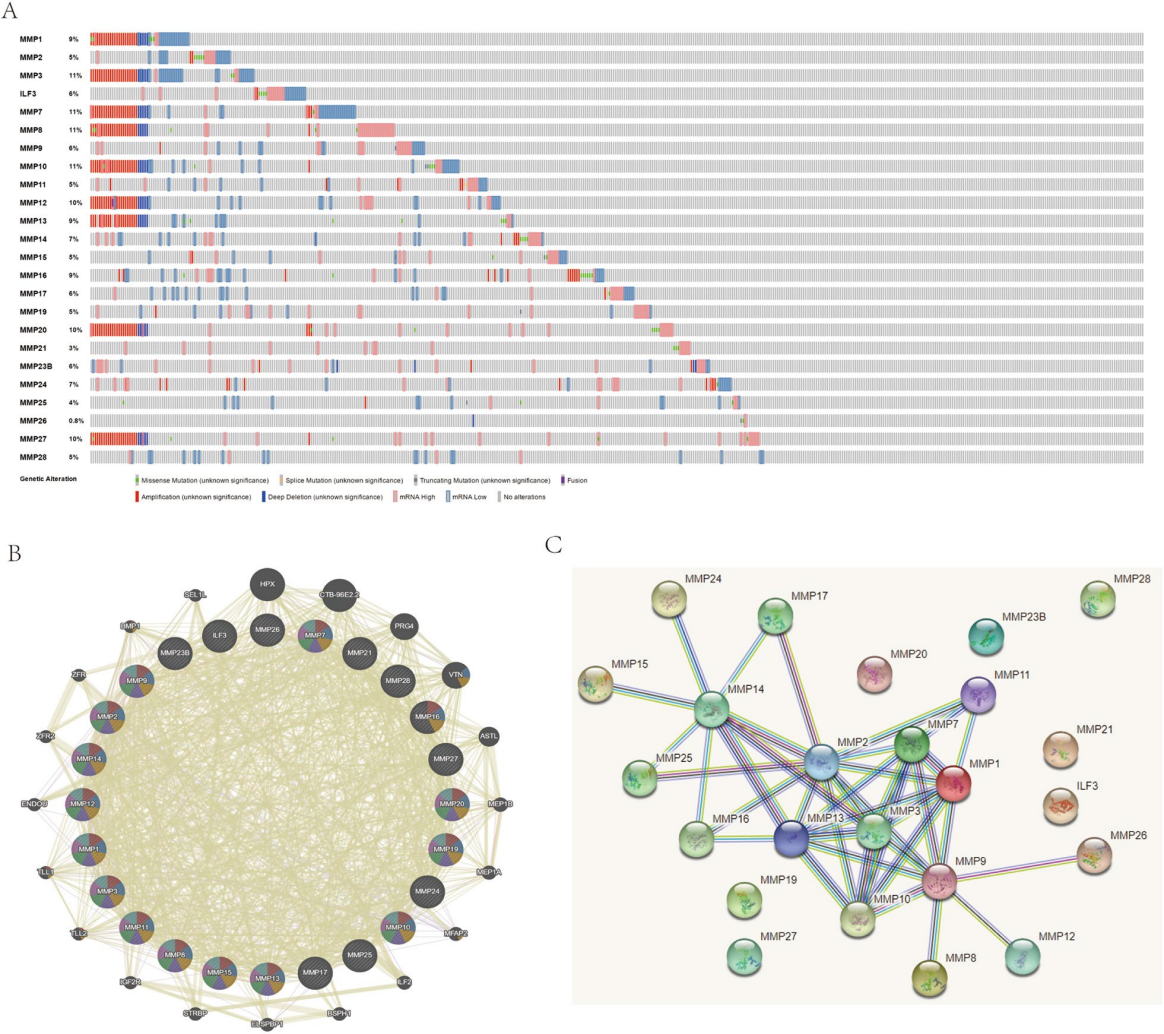
Genetic changes, adjacent gene networks and interaction analysis of the MMP family in patients with head and neck squamous cell carcinoma **A** GENETIC changes of the MMP family in HNSC; **B**, **C** PPI network of the MMP family

The genetic changes of the MMP family gene were analyzed and described as the tumors with mutation, amplification, deep deletion, high mRNA level and multiple changes. MMP1, MMP3, MMP7, MMP8, MMP10, MMP12, MMP13, MMP20 and MMP27 all had ≥ 9% genetic changes. Furthermore, the amplification and depth deletion were greater, while the other MMP had only minor genetic changes (Fig. 7A). GeneMANIA was used to analyze the correlation of the MMP family and its adjacent genes at gene level, found that MMP1, MMP2, MMP3, ILF3, MMP7, MMP8, MMP9, MMP10, MMP11, MMP12, MMP13, MMP14, MMP15, MMP16, MMP17, MMP19, MMP20, MMP21, MMP23B, BMMP24, MMP25, MMP26, MMP27, MMP28 were closely related to HPX, CTB-96E2.2, PRG4, VTN, ASTL, MEP1B, MEP1A, MFAP2, ILF2, BSPH1, ELSPBP1, STRBP, IEF2R, TLL2, TLL1, ENDOU, ZFR2, ZFR, BMP1, SEL1L (Fig. 7B). The interaction at the expression level of MMP protein was determined by STRING analysis. In this analysis, other MMP proteins except ILF3, MMP19, MMP20, MMP21, MMP23B, MMP27, and MMP28 interacted (Fig. 7C).

### Function enrichment of the MMP family and the role of related signal pathways

**Fig. 8:**
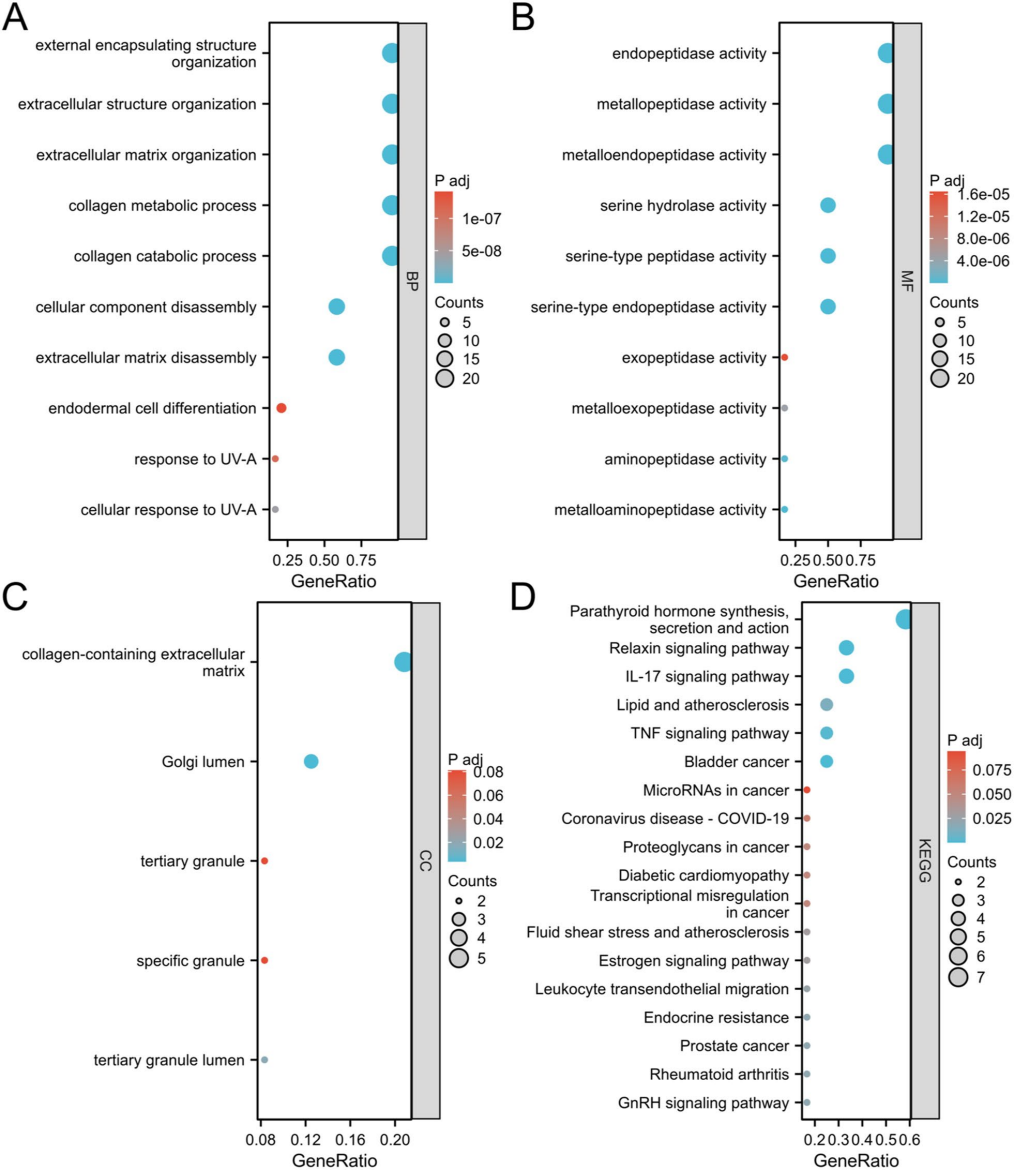
The enrichment analysis of the MMP family in HNSC. **A** Bar plot of GO enrichment in BP terms. **B** Bar plot of GO enrichment in CC terms. **C** Bar plot of GO enrichment in MF terms. **D** Bar plot of KEGG enriched terms

For a deeper understanding of the MMP family, GO enrichment analysis and KEGG pathway enrichment analysis of the MMP family and adjacent genes were isolated from DAVID6.8 in this study. GO enrichment analysis included BP, CC, MF. As shown in Fig. 8, the MMP family and adjacent genes in BP were the most enriched in the process of collagen metabolism and collagen catabolism of extracellular structures and tissues of extracellular matrix, and the enrichment was most significant in gastral action (Fig. 8A). In MF, the number of genes enriched in the activities of endopeptidase, metallopeptidase and endometal peptidase increased and the enrichment was significant in the activity of exopeptidase (Fig. 8B). Genes in CC were enriched in the collagen-containing extracellular matrix (Fig. 8C). KEGG analysis showed that a large number of these genes were enriched in the synthesis and secretion of parathyroid hormone, and were significantly enriched in the transcriptional regulation of cancer (Fig. 8D).

### Transcription factor targets and kinase targets of the MMP family in head and neck squamous cell carcinoma

MMP1, MMP2, MMP3, MMP7, MMP9, MMP10, MMP11, MMP12, MMP13, MMP14, MMP17, MMP20, and MMP28 were contained in TRUST. We found that 21 transcription factors were related to the regulation of the MMP family chemokines. JUN was a key transcription factor of MMP1, MMP2, MMP3, MMP7, MMP9, MMP12, MMP13, and MMP20; STAT3 was a key transcription factor of MMP1, MMP2, MMP7, MMP9, MMP10, and MMP14; ETV4 was a key transcription factor of MMP1, MMP2, MMP7, and MMP14; ETS1 was a key transcription factor of MMP1, MMP3, MMP9, MMP10, MMP13; RELA was a key transcription factor of MMP1, MMP2, MMP3, MMP9, MMP12, MMP13, MMP14; ETS2 was a key transcription factor of MMP1, MMP2, MMP3 and MMP9. MAZ was a key transcription factor of MMP1, MMP9 and MMP14. FOS was a key transcription factor of MMP1, MMP3, MMP7 and MMP9. NFKB1 was a key transcription factor of MMP1, MMP2, MMP3, MMP9, MMP13, MMP14; NFKBIA was a key transcription factor of MMP1, MMP3 and MMP9; SRF was a key transcription factor of MMP2, MMP9 and MMP14; NCOA3 was a key transcription factor of MMP7 and MMP10; KLF8 was a key transcription factor of MMP9 and MMP14; SNAI2 was a key transcription factor of MMP9 and MMP17; SP1 was a key transcription factor of MMP2, MMP9, MMP11 MMP14, MMP28; RUNX2 was a key transcription factor of MMP2 and MMP13; CTNNB1 was a key transcription factor of MMP7 and MMP14; YBX1 was a key transcription factor of MMP2 and MMP13. Both TWSIT1 and TP53 were key transcription factors of MMP1 and MMP2; PPARG was a key transcription factor of MMP1 and MMP9; HDAC1 was a key transcription factor of MMP9 and MMP28; TFAP2A was a key transcription factor of MMP2 and MMP9; STAT1 was a key transcription factor of MMP9 and MMP13 (Table 2).

**Table 2:** Key regulated factor of the MMP family in HNSC

Key TF	Description	Regulated gene	*P* value	FDR
JUN	jun proto-oncogene	MMP1, MMP2, MMP3, MMP7, MMP9, MMP12, MMP13, MMP20	8.23e−12	1.97e−10
STAT3	signal transducer and activator of transcription 3 (acute-phase response factor)	MMP1, MMP2, MMP3, MMP7, MMP9, MMP10, MMP14	3.65e−10	4.38e−09
ETV4	ets variant 4	MMP1, MMP2, MMP7, MMP14	1.45e−08	1.16e−07
ETS1	v-ets erythroblastosis virus E26 oncogene homolog 1 (avian)	MMP1, MMP3, MMP9, MMP10, MMP13	4.51e−08	2.71e−07
RELA	v-rel reticuloendotheliosis viral oncogene homolog A (avian)	MMP1, MMP2, MMP3, MMP9, MMP12, MMP13, MMP14	6.7e−08	2.82e−07
ETS2	v-ets erythroblastosis virus E26 oncogene homolog 2 (avian)	MMP1, MMP2, MMP3, MMP9	7.06e−08	2.82e−07
MAZ	MYC-associated zinc finger protein (purine-binding transcription factor)	MMP1, MMP9, MMP14	1.01e−07	3.46e−07
FOS	FBJ murine osteosarcoma viral oncogene homolog	MMP1, MMP3, MMP7, MMP9	7.59e−07	2.28e−06
NFKB1	nuclear factor of kappa light polypeptide gene enhancer in B-cells 1	MMP1, MMP2, MMP3, MMP9, MMP13, MMP14	1.72e−06	4.15e−06
NFKBIA	nuclear factor of kappa light polypeptide gene enhancer in B-cells inhibitor, alpha	MMP1, MMP3, MMP9	1.73e−06	4.15e−06
SRF	serum response factor (c-fos serum response element-binding transcription factor)	MMP2, MMP9, MMP14	4.08e−06	8.9e−06
NCOA3	Nuclear receptor coactivator 3	MMP7, MMP10	3.24e−05	6.49e−05
KLF8	Kruppel-like factor 8	MMP9,MMP14	4.32e−05	7.98e−05
SNAI2	snail homolog 2 (Drosophila)	MMP9, MMP17	1.40e−04	2.40e−04
SP1	Sp1 transcription factor	MMP2, MMP9, MMP11, MMP14, MMP28	2.75e−04	4.12e−04
RUNX2	runt-related transcription factor 2	MMP2, MMP13	2.62e−04	4.12e−04
CTNNB1	catenin (cadherin-associated protein), beta 1, 88 kDa	MMP7, MMP14	3.53e−04	4.98e−04
YBX1	Y box binding protein 12	MMP2, MMP13	6.60e−04	8.8e−04
TWIST1	twist basic helix-loop-helix transcription factor 1	MMP1, MMP2	8.99e−04	0.001
PPARG	peroxisome proliferator-activated receptor gamma	MMP1, MMP9	0.003	0.004
HDAC1	histone deacetylase 1	MMP9, MMP28	0.004	0.004
TFAP2A	transcription factor AP-2 alpha (activating enhancer binding protein 2 alpha) 2	MMP2, MMP9	0.004	0.004
STAT1	signal transducer and activator of transcription 1, 91 kDa	MMP9, MMP13	0.005	0.005
TP53	tumor protein p53	MMP1, MMP2	0.018	0.018

We identified the first two kinase targets of the MMP family from the LinkedOmics database. ATR and ATM were the most common first two kinase targets in the MMP family. ATR and ATM kinase targets were found in the first two kinases of MMP1, MMP2, MMP3, ILF3, MMP9, MMP10, MMP11, MMP13, MMP15, MMP17, MMP23B and MMP24 (Table 3).

**Table 3:** The Kinase target networks of the MMP family in HNSC

MMP	Enriched kinase target	Description	Leading EdgeNum	*P* value
MMP1	CDK2	Cyclin dependent kinase 2	99	0.000
	ATM	ATM serine/threonine kinase	51	0.000
MMP2	ATR	ATR serine/threonine kinase	28	0.000
	NEK2	NIMA related kinase 2	5	0.000
MMP3	ROCK1	Rho associated coiled-coil containing protein kinase 1	19	0.000
	ATR	ATR serine/threonine kinase	34	0.000
ILF3	ATR	ATR serine/threonine kinase	33	0.000
	PLK1	Polo like kinase 1	33	0.000
MMP7	MAP3K5	Mitogen-activated protein kinase kinase kinase 5	7	0.000
	PKN2	Protein kinase N2	7	0.000
MMP8	CDK5	Cyclin dependent kinase 5	30	0.002
	NTRK1	Neurotrophic receptor tyrosine kinase 1	7	0.003
MMP9	ATM	ATM serine/threonine kinase	53	0.000
	ATR	ATR serine/threonine kinase	32	0.000
MMP10	ROCK1	Rho associated coiled-coil containing protein kinase 1	23	0.011
	ATM	ATM serine/threonine kinase	45	0.000
MMP11	ATR	ATR serine/threonine kinase	33	0.000
	AURKB	Aurora kinase B	32	0.000
MMP12	LYN	LYN proto-oncogene, Src family tyrosine kinase	20	0.000
	SYK	Spleen associated tyrosine kinase	14	0.000
MMP13	ATM	ATM serine/threonine kinase	37	0.000
	ATR	ATR serine/threonine kinase	33	0.000
MMP14	PRKCG	Protein kinase C gamma	11	0.000
	ROCK1	Rho associated coiled-coil containing protein kinase 1	18	0.000
MMP15	CDK1	Cyclin dependent kinase 1	79	0.000
	ATR	ATR serine/threonine kinase	21	0.000
MMP16	FYN	FYN proto-oncogene, Src family tyrosine kinase	31	0.019
	CDK5	Cyclin dependent kinase 5	25	0.000
MMP17	ATM	ATM serine/threonine kinase	49	0.000
	ATR	ATR serine/threonine kinase	35	0.000
MMP19	MAP2K4	Mitogen-activated protein kinase kinase 4	4	0.000
	LYN	LYN proto-oncogene, Src family tyrosine kinase	20	0.000
MMP20	PKN2	Protein kinase N2	6	0.000
	ZAP70	Zeta chain of T-cell receptor associated protein kinase 70	8	0.000
MMP21	GRK4	G protein-coupled receptor kinase 4	2	0.007
	MKNK2	MAP kinase interacting serine/threonine kinase 2	3	0.035
MMP23B	ATM	ATM serine/threonine kinase	57	0.000
	MAP3K8	Mitogen-activated protein kinase kinase kinase 8	11	0.000
MMP24	CDK1	Cyclin dependent kinase 1	108	0.000
	ATM	ATM serine/threonine kinase	56	0.000
MMP25	FYN	FYN proto-oncogene, Src family tyrosine kinase	21	0.000
	SYK	Spleen associated tyrosine kinase	16	0.000
MMP26	LCK	LCK proto-oncogene, Src family tyrosine kinase	28	0.000
	JAK2	Janus kinase 2	11	0.011
MMP27	CHEK1	Checkpoint kinase 1	48	0.000
	PLK1	Polo-like kinase 1	34	0.000
MMP28	LCK	LCK proto-oncogene, Src family tyrosine kinase	21	0.000
	PRKD1	Protein kinase D1	10	0.000

**Fig. 9:**
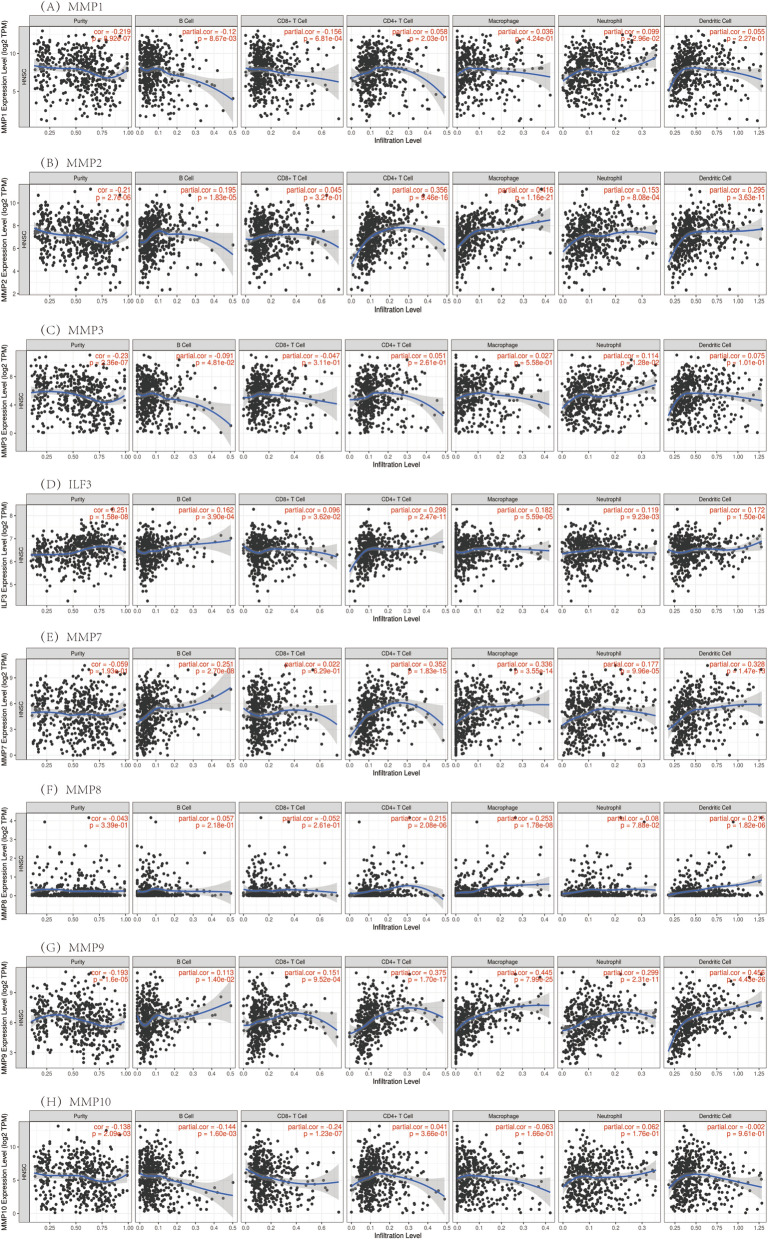

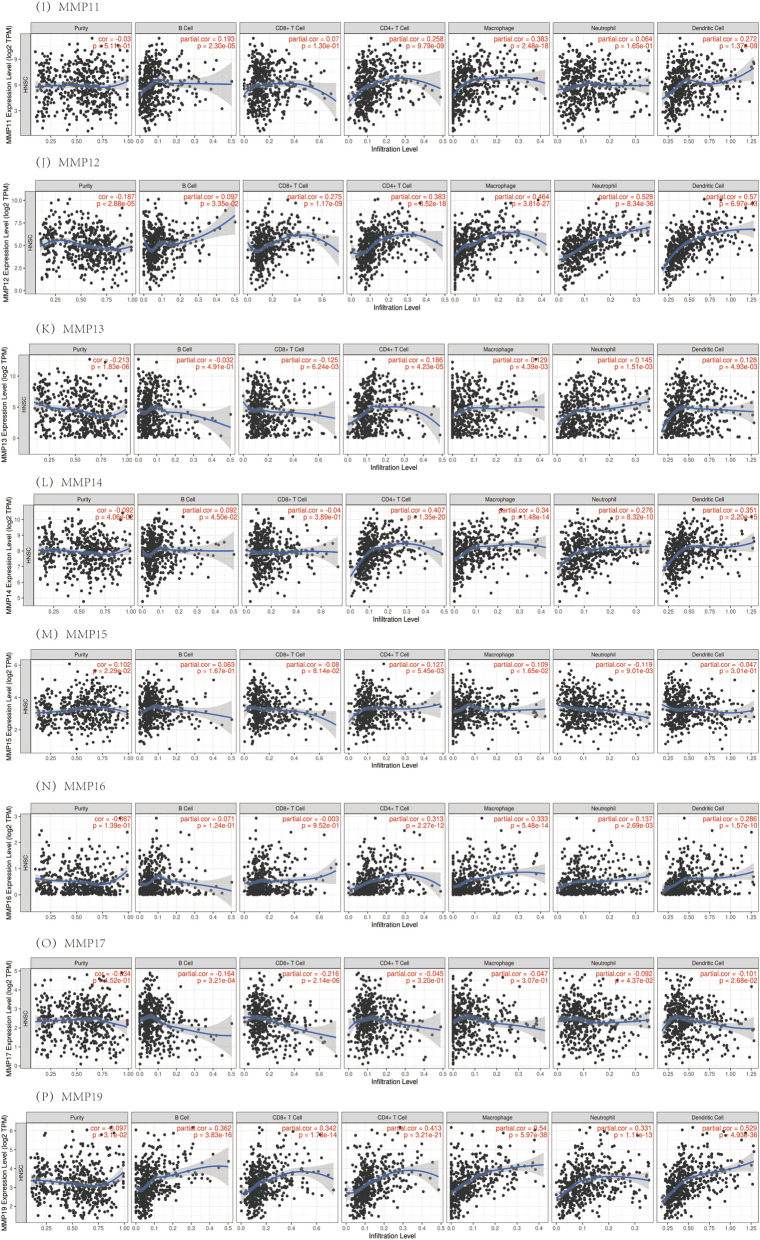

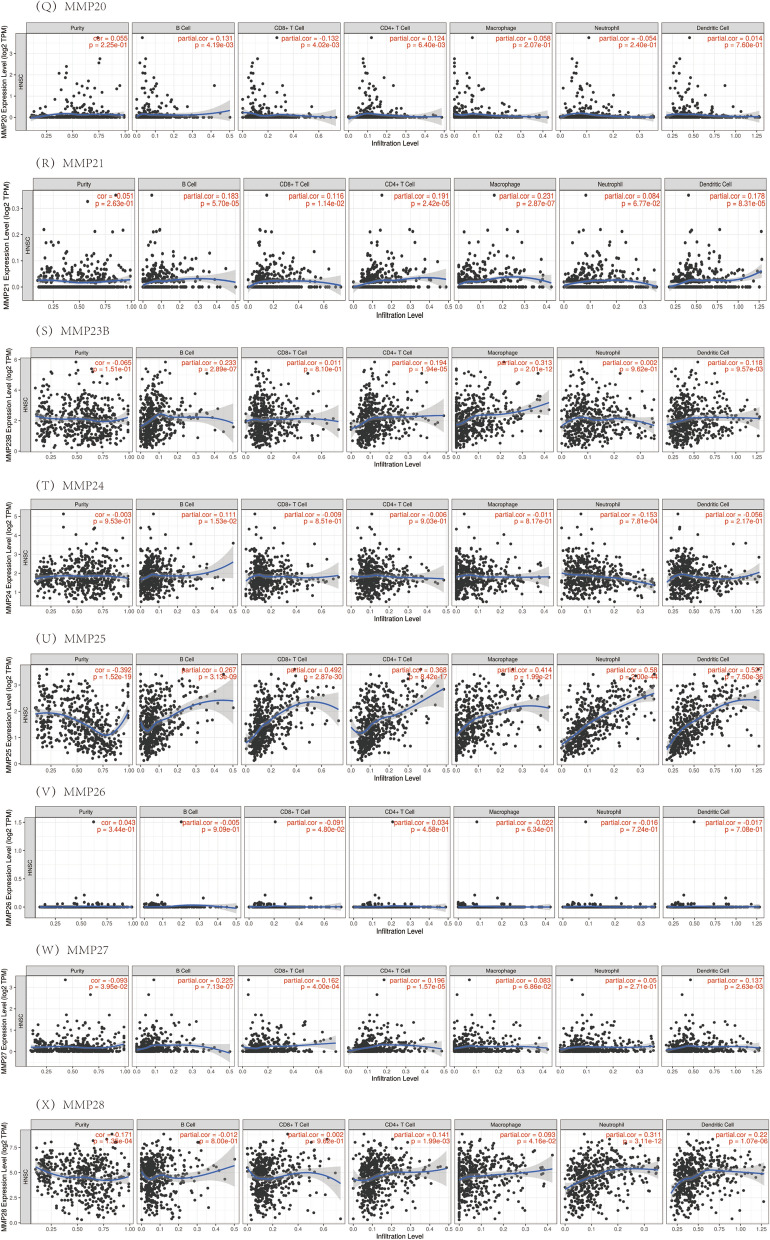
The correlation between the MMP family and immune cell infiltration

In this study, the gene module was used to evaluate the correlation between the MMP family levels and immune cell infiltration. The survival module was used to evaluate the correlation between clinical outcomes and immune cell infiltration and the MMP family. The expression level of MMP1 was negatively correlated with B cells and CD8 + T cells, positively correlated with neutrophil infiltration level, and not significantly correlated with CD4 + T cells, macrophages and dendritic cells infiltration level. The expression levels of MMP2, MMP7 and MMP11 had no significant correlation with the infiltration levels of CD8 + T cells, but had a significant positive correlation with the infiltration levels of other immune cells. The expression level of MMP3 was positively correlated with neutrophil infiltration level. It was found that ILF3, MMP9, MMP12, MMP19, MMP25 were significantly positively correlated with the levels of immune cells. MMP8 was significantly positively correlated with the levels of infiltration of CD4 + T cells, macrophages and dendritic cells. The expression of MMP10 was negatively correlated with B cells and CD8 + T. The expression level of MMP13 was negatively correlated with CD8 + T cells, positively correlated with the infiltration level of CD4 + T cells, macrophages, neutrophils and dendritic cells, but not significantly correlated with the infiltration level of B cells. MMP14 and MMP16 showed significant positive correlation with the infiltration levels of CD4 + T cells, macrophages, neutrophils and dendritic cells, but no correlation with other immune cells. MMP15 was negatively correlated with neutrophil infiltration level, positively correlated with CD4 + T cells and macrophages infiltration level, and had no correlation with B cells, CD8 + T cells and dendritic cell immune cells. MMP17 was negatively correlated with B cells, CD8 + T cells and dendritic cells, but had no correlation with other immune cells. MMP20 was negatively correlated with CD8 + T cells, positively correlated with B cells and CD4 + T cells, and had no correlation with other immune cells. There was no correlation between MMP21 and neutrophil infiltration, but a significant positive correlation between MMP21 and other immune cells. MMP23B was significantly positively correlated with the infiltration levels of B cells, CD4 + T cells, macrophages and dendritic cells, while the other immune cells had no correlation. MMP24 was negatively correlated with neutrophil infiltration level, positively correlated with B cell infiltration level, and had no correlation with other immune cells. There was no correlation between MMP26 and the levels of immune cell infiltration. MMP27 was not correlated with macrophages and neutrophils, but positively correlated with other immune cells. MMP28 was positively correlated with the infiltration levels of CD4 + T cells, neutrophils and dendritic cells, but not with other immune cells. The results showed that the influence of gene expression on the microscopic characterization of immune infiltration was extremely complex and variable, reflecting the heterogeneity and complexity of the immune microenvironment (Fig. 9).

Cox proportional risk model was used and the following confounders were corrected: B cells, CD8 + T, CD4 + T cells, neutrophils, dendritic cells, MMP1, MMP2, MMP3, ILF3, MMP7, MMP8, MMP9, MMP10, MMP11, MMP12, MMP13, MMP15, MMP17, MMP20, MMP21, MMP23B, MMP24, MMP25, MMP 26, MMP27, MMP28, macrophages, MMP14, MMP16 and MMP19 were significantly associated with clinical outcomes in HNSC patients (Table 4).

**Table 4:** The cox proportional hazard model of the MMP family faiand six tumor infitrating immune cells in HNSC

	Coef	HR	95%CI_l	95%CI_u	*P* value	sig
B_cell	− 1.333	0.264	0.015	4.682	0.364	
CD8_Tcell	− 0.500	0.607	0.068	5.406	0.654	
CD4_Tcell	− 3.189	0.041	0.001	1.499	0.082	
Macrophage	3.614	37.115	1.702	809.421	0.022	*
Neutrophil	0.687	1.988	0.047	84.929	0.720	
Dendritic	0.753	2.123	0.271	16.613	0.473	
MMP1	0.061	1.063	0.909	1.242	0.446	
MMP2	− 0.005	0.995	0.803	1.233	0.967	
MMP3	0.012	1.012	0.913	1.123	0.815	
ILF3	− 0.246	0.782	0.560	1.091	0.148	
MMP7	0.015	1.015	0.927	1.112	0.742	
MMP8	0.201	1.223	0.854	1.751	0.272	
MMP9	0.054	1.055	0.931	1.197	0.401	
MMP10	− 0.006	0.994	0.900	1.098	0.908	
MMP11	0.004	1.004	0.898	1.122	0.948	
MMP12	− 0.087	0.917	0.804	1.046	0.195	
MMP13	− 0.023	0.977	0.902	1.058	0.565	
MMP14	0.607	1.836	1.363	2.473	0.000	***
MMP15	0.027	1.027	0.845	1.249	0.789	
MMP16	− 0.869	0.419	0.264	0.665	0.000	***
MMP17	− 0.186	0.830	0.675	1.022	0.079	
MMP19	− 0.455	0.635	0.478	0.842	0.002	**
MMP20	0.252	1.286	0.903	1.833	0.164	
MMP21	1.838	6.284	0.084	470.568	0.404	
MMP23B	0.054	1.056	0.886	1.258	0.542	
MMP24	− 0.076	0.927	0.739	1.162	0.510	
MMP25	− 0.275	0.759	0.571	1.010	0.058	
MMP26	− 5.121	0.006	0.000	68.064	0.283	
MMP27	− 0.034	0.967	0.606	1.541	0.886	
MMP28	− 0.045	0.956	0.855	1.068	0.423	

^*^P < 0.05, **P < 0.01, ***P < 0.001

## Discussion

Matrix metalloproteinase is a Zn-dependent protease that has been shown to degrade the extracellular matrix. In recent years, a large number of studies have shown that it played a significant regulatory role in tumor cell invasion, proliferation, metastasis, immunity and angiogenesis [26, 27]. The MMP family played an important role in the whole process of disease occurrence and tumor development, and studying how it participates in different stages of cancer may help to develop a specific therapy [28]. At present, many researchers have studied the role of the MMP family in the occurrence and development of HNSC. ACY-241 and JQ1 have been found to regulate MMP-2 and MMP-9 expression via the TNF-/AKT/NF-B axis and to synergistically inhibit HNSC metastasis synergistically [29]. However, the study of the MMP family as a therapeutic target and prognostic biomarker in HNSC has not been clear and systematic. Firstly, we discussed the mRNA expression level of the MMP family in various tumors and different HNSC and its relationship with pathological stage. We found that the expression levels of MMP1, MMP3, ILF3, MMP7, MMP9, MMP10, MMP11, MMP12, MMP13 and MMP16 in most tumor tissues were higher than those in normal tissues, while the expression levels of MMP15 were lower. With the development of HNSC, the expression of MMP3 and MMP11 increased, and the expression of MMP25 decreased significantly. These data indicate that the MMP family plays an important role in the occurrence and development of HNSC. Chunwen Su [30] showed that MMP3 could be used as a potential biomarker of oral cancer progression. Further, we investigated the prognostic value of the MMP family in HNSC patients and found that the MMP family transcription levels were not significantly correlated with disease-free survival, while MMP1, MMP8, and MMP25 were significantly correlated with overall survival. Kun Wu [31] found that urokinase-type plasminogen activator (PLAU1) regulates the expression of MMP1 in HNSC, thereby affecting the proliferation, invasion and metastasis of HNSC. Therefore, PLAU1 may be a potential therapeutic target for HNSC. In HPV-negative squamous cell oropharyngeal carcinoma patients, high serum level of matrix metalloproteinase inhibitor (TIMP-1) is associated with poor OS and DFS, suggesting that high serum level of TIMP-1 is associated with poor prognosis in HPV-negative squamous cell oropharyngeal carcinoma patients [32]. MMP-7 expression may affect the distal recurrence rate and disease-specific survival rate of HPV-positive oropharyngeal squamous cell carcinoma [33]. However, previous studies on the expression level and prognostic value of the MMP family in HNSC have been limited.

In this study, GeneMANIA was used to conduct correlation analysis of the MMP family and its adjacent genes and STRING analysis, and the MMP family were determined to correlate with 20 adjacent genes. Besides ILF3, MMP19, MMP20, MMP21, MMP23B, MMP27 and MMP28, Interaction of other MMP at the protein expression level. In this analysis, other MMP proteins except ILF3, MMP19, MMP20, MMP21, MMP23B, MMP27, and MMP28 interacted with each other. Based on TCGA database, genetic changes of the MMP family were obtained from cBiopartal. All the MMP family members had gene mutations, among which MMP1, MMP3, MMP7, MMP8, MMP10, MMP12, MMP13, MMP20 and MMP27 all had ≥ 9% mutations. There were more amplifications and depth loss. Studies have shown that MMP-7 gene promoter (181 A/G) and MMP-9 (-1562 C/T) polymorphisms were significantly correlated in oral tongue squamous cell carcinoma (OTSCC) [34]. Gene polymorphisms of MMP1, MMP2, MMP9, MMP11 and MMP13 were significantly correlated with HNSC [35]. Gene mutations play an important role in the complex process of HNSC occurrence and progression.

Then, we focused on the function of the MMP family by GO enrichment analysis and KEGG pathway enrichment analysis. We found that the MMP family enriched in the extracellular matrix, and the adjacent genes most endopeptidase activity within the peptide enzyme activity, metal, metal enrichment significantly on peptide enzyme activity such as function, in the process of collagen and collagen protein catabolism metabolic process of enrichment, suggesting that the MMP family can exist in the extracellular matrix, can be adjusted by parathyroid hormone peptide enzyme activity, and it can participate in the catabolic process of collagen.

We explored the transcription factor targets and kinase targets of the MMP family in HNSC, and found that MMP1, MMP2, MMP3, MMP7, MMP9, MMP10, MMP11, MMP12, MMP13, MMP14, MMP17, MMP20, and MMP28 were contained in TRUST. We found that 21 transcription factors were associated with the regulation of the MMP family chemokines. JUN, STAT3, EST1, RELT and NFKB1 were common key transcription factors in MMP. Weiyi Wang [36] found that dihydroartemisinin could inhibit STAT3 activation, down-regulate MMP-9, and affect the invasion and metastasis of cancer stem cells (CSCs) in laryngeal cancer. Licorice chalcione D (LCD) can inhibit the expression of P-JAK2 and P-STAT3 and induce the expression of caspase 3, which can be used for treating OSCC [37]. ATR and ATM were the most common kinase targets in the MMP family. ATR and ATM kinase targets can be seen in the first two kinases of MMP1, MMP2, MMP3, ILF3, MMP9, MMP10, MMP11, MMP13, MMP15, MMP17, MMP23B and MMP24. Therefore, kinase inhibitors targeting kinase targets were one direction for the treatment of HNSC. Vendetti FP ATR kinase inhibitor AZD6738 blocks PD-L1 upregulation in tumor cells and significantly reduces the number of tumor infiltration-regulating cells [38]. Faulhaber EM [39] showed that DNA-PK, ATM, and ATR kinase inhibitors combined with ionizing radiation can increase HNSC tumor cell death while preserving normal tissue cells.

In this study, we found that the expression of the MMP family correlated with the infiltration levels of six immune cells, B cells, CD8 + T cells, CD4 + T cells, macrophages, neutrophils, and dendritic cells, suggesting that the MMP family could reflect the immune status and serve as a prognostic indicator. The infiltration levels of macrophages, neutrophils and dendritic cells were correlated, indicating that the MMP family could reflect the immune status and serve as a prognostic indicator. Cox proportional risk model analysis showed that macrophages, MMP14, MMP16 and MMP19 were significantly correlated with the clinical prognosis of HNSC patients.

## Conclusion

This study systematically analyzed the role and feasibility of members, which were used as the therapeutic targets and prognostic biomarkers of the MMP family, to find new targets for future drug development of HNSC, and provide a systematic prognostic model of the MMP family for patient survival analysis. In our future study, the expression levels of members of the MMP family that can be used as therapeutic targets and prognostic biomarkers for HNSC will be experimentally verified, and comprehensively explore the regulatory relationships of interacting genes and proteins with these therapeutic targets and prognostic biomarkers, meanwhile, the enrichment analysis and the regulatory relationship of transcription factors of the significance targets and biomarkers will be further discussed, to find their regulatory relationship in the signaling pathway, in order to find the mechanism of the MMP family members as therapeutic targets and prognostic biomarkers in the microenvironment of HNSC systematically and comprehensively.

## Data Availability

The original data presented in this study are included in the paper.
